# Phacoemulsification Tip Elongation Under Standardized Resistance: An Objective Measure of Human Crystalline Lens Hardness

**DOI:** 10.1167/tvst.12.4.6

**Published:** 2023-04-05

**Authors:** Tommaso Rossi, Mario R. Romano, Antonio Carotenuto, Carlo Malvasi, Giov Battista Angelini, Alessandro Rossi, Serena Telani, Guido Ripandelli, Giorgio Querzoli

**Affiliations:** 1 IRCCS Fondazione Bietti ONLUS, Roma, Italy; 2 Department of Biomedical Science, Humanitas University, Milan, Italy; 3 Optikon 2000 SpA, Rome, Italy; 4 IRCCS Policlinico San Martino, Genova, Italy; 5 DICAAR, Università di Cagliari, Cagliari, Italy

**Keywords:** cataract surgery, phacoemulsification, crystalline lens hardness

## Abstract

**Purpose:**

To establish a correlation between phacoemulsification tip normalized driving voltage (NDV) and crystalline lens hardness and use it as an objective measure of lens hardness. The study used a phaco tip equipped with previously validated elongation control adjusting the driving voltage (DV) to produce invariant elongation regardless of resistance.

**Methods:**

The laboratory study measured the mean and maximum DV of the phaco tip immersed in glycerol–balanced salt solution and correlated the DV with the kinematic viscosity at 25, 50, and 75 µm tip elongation. The NDV were obtained by dividing the DV in glycerol by the DV in the balanced salt solution. The clinical arm of the study recorded DV of 20 consecutive cataract surgeries. The correlation of mean and maximum NDV to Lens Opacities Classification System (LOCS) III classification, patient's age and effective phaco time were evaluated.

**Results:**

The mean and maximum NDV correlated with the kinematic viscosity of the glycerol solution (*P* < 0.001 in all cases). Mean and maximum NDV during cataract surgery correlated with patients’ age, effective phaco time, LOCS III nuclear color, and nuclear opalescence (*P* < 0.001 in all cases).

**Conclusions:**

When a feedback algorithm is running, DV variation strictly correlates with encountered resistance in glycerol solutions and real-life surgery. NDV significantly correlates with the LOCS classification. Future developments might include sensing tips that react to lens hardness in real time.

**Translational Relevance:**

The study correlates for the first time phaco tip DV and crystalline lens mechanical properties, establishing an objective and reliable measure of lens hardness. This may lead to smart phaco tips reacting to cataract hardness change in real time and sparing ultrasound dispersion.

## Introduction

Phacoemulsification is the standard of care for cataract surgery, the most prevalent of all surgical procedures at more than 20 million cases in the world per year. During surgery, crystalline lens material is emulsified through the jackhammer-like action of the phacoemulsificator (called the “phaco” from now on) hollow cylindric tip oscillating at ultrasonic frequency and amplitudes (i.e., elongation) usually comprised between 0 and 100 µm.

As the phaco tip digs into the lens cortex and nucleus material, the lens resistance to tip penetration varies continuously owing to the layered crystalline structure and as a function of mechanical properties related to age and countless other variables. Despite numerous attempts to measure consistently cataract “hardness” (i.e., resistance to the phaco tip penetration),^1^ most proposed methods rely on characteristics only marginally and indirectly related to lens mechanical properties,^2^ such as subjective or objective^3^ optical transparency^4^ or color comparison with predetermined scales which became the clinical standard (Lens Opacities Classification System [LOCS III]),^5^ although ultrasonic attenuation has also been proposed.^6^

Phaco tip oscillation is generated by piezoelectric crystals in response to driving voltage (DV): harder nuclei offer greater resistance to penetration and require a higher voltage to attain a given tip elongation. We previously described the accuracy of a patented feedback mechanism using the direct piezoelectric effect to monitor in real-time the tip elongation and adjust the DV according to encountered resistance to ensure the prescribed elongation irrespective of lens hardness variation.^7^

**Figure 1.**
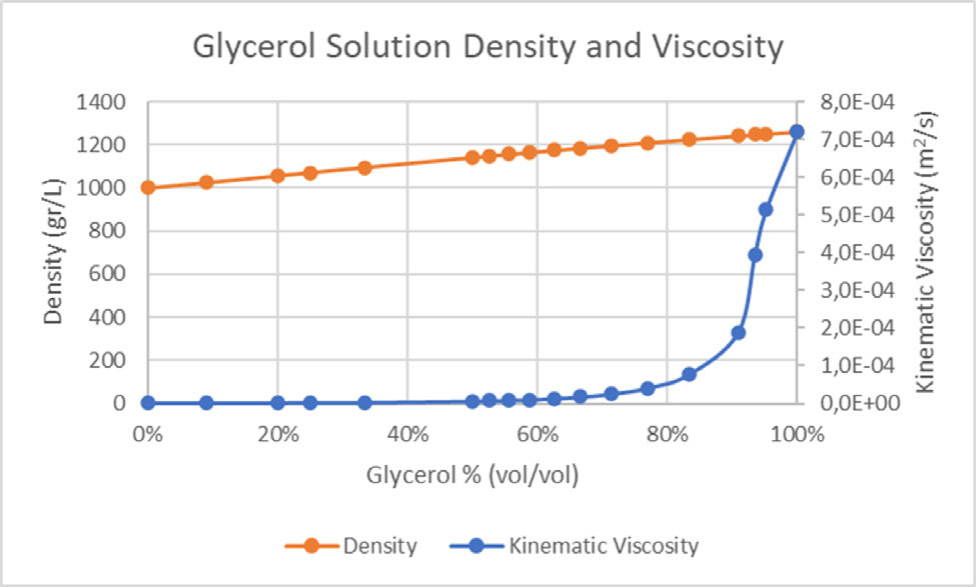
Density and viscosity of increasingly pure glycerol solutions. Note the steep increase of kinematic viscosity curve between 80% and 100% (pure glycerol).

Glycerol and water solutions are often used as reference in hydraulic engineering since they retain well-known physical properties and kinematic viscosity spanning four orders of magnitude as volume/volume percentage ranges from 0% (pure water) to 100% (pure glycerol)^8^ (Fig. 1).

The purpose of present paper is, first, to investigate in the laboratory whether there is a correlation between phaco tip DV and the resistance offered by standardized and increasingly viscous glycerol solutions, compared with a balanced salt solution (BSS). Second, transpose the validated results to the operating room to assess objectively crystalline lens hardness during surgery as a function of DV required to keep the desired phaco tip elongation during lens emulsification. Third, the study evaluates the correlation between LOCS III lens grading systems,^9^^,^^10^ effective phaco time (EPT), and encountered resistance during phacoemulsification.

## Methods

### Technical Background

Piezoelectric crystals (also named “ceramics”) deform when exposed to electric potential difference (indirect piezoelectric effect) and, conversely, polarize, generating an electric tension when deformed (direct piezoelectric effect). Ceramics can, therefore, alternatively drive phaco tip as a function of applied voltage or generate voltage as a function their own elongation (distortion) if DV is temporarily halted. In the latter case, the resulting direct piezoelectric voltage (PV) is strictly proportional to deformation for a given crystal and therefore represents an accurate measure of actual, real-time phaco tip elongation.

The minimal stress patent (from here on denominated “the feedback control system” or “the control system”) is a continuous feedback control of tip elongation (US6175180B1) described in detail elsewhere.^7^ Briefly, the feedback mechanism periodically interrupts DV to the phaco tip for 250 µs at 100 Hz (i.e., 100 times per second) and records deformation-generated voltage to calculate actual elongation (hereafter referred to as PV). If there is a mismatch between the prescribed and the actual elongation, the DV is adjusted accordingly.

### Glycerol Solution

Glycerol/water solutions were used as a standardized way to oppose known, incremental resistance to phaco tip oscillation and characterize required tension as a function of kinematic viscosity ranging from 1.0E-6 m^2^/s (0% glycerol; i.e., pure water) to 1.1E-3 m^2^/s (100% or pure glycerol) (Fig. 1) at 20°C. Glycerol solutions were prepared at 20°C as shown in Table 1; chosen dilutions were distributed among the entire sigmoid curve to adequately represent especially the steepest tract of the function between 80% and 100% glycerol volume (Fig. 1).

**Table 1.** Physical Properties of Glycerol Solution

Temperature (°C)	Glycerol (mL)	H_2_O (mL)	% (vol/vol)	Kinematic Viscosity (m^2^/s)	Density (g/L)
20	10	0	100.00	7.20E-04	1260.8
20	10	0.5	95.24	5.15E-04	1251.3
20	10	0.7	93.46	3.95E-04	1247.5
20	10	1	90.91	1.89E-04	1242.0
20	10	2	83.33	7.74E-05	1225.0
20	10	3	76.92	4.09E-05	1210.0
20	10	4	71.43	2.53E-05	1196.9
20	10	5	66.67	1.74E-05	1185.2
20	10	6	62.50	1.29E-05	1174.7
20	10	10	50.00	6.02E-06	1142.0
20	5	15	25.00	1.96E-06	1071.5
20	0	1	0.00	9.00E-07	1000.0

### Experimental Setting

A Revo Smart phacovitrectomy machine equipped with Minimal Stress patent (Optikon 2000 Inc., Rome, Italy) was used in all measures both in the laboratory and in the surgical theatre. The Revo Smart had been modified to record DV and ceramic elongation voltage used by the feedback control in real-time, at 100 Hz (100 readings per second). Data were saved in .csv files and then converted to .xls for analysis and statistical purposes. The same phacoemulsification handpiece (Optikon 2000 Inc.) was used in all laboratory measures after the proper priming and tuning procedure, equipped with a flared, 20G, 30° beveled tip.

### Laboratory Measures

The phaco tip was immersed into 30 mL of the selected solution at 20°C and activated for 2 seconds while the temperature, phaco tip DV, and PV were recorded (yielding 200 data points at 100 Hz). Ultrasound waves were delivered in a continuous mode at 40 KHz and phaco tip elongation preset at 25, 50, and 75 microns for each solution. Each measure was repeated three times and average mean and maximum voltage values were considered.

Because glycerol solution viscosity and density change as a function of temperature, a thermo-couple with 0.1°C resolution (0.2 mm in diameter, type K; Fluke Corp., Everett, WA) was applied close to phaco tip distal end to record temperature throughout measure sessions. All measures started at the same baseline 20°C temperature.

A volume pump recirculated the glycerol solution generating a continuous flow on top of the phaco tip to reduce any increase in temperature. In all cases, the glycerol solution viscosity was calculated based on the phaco tip actual temperature reached during that specific measure, however, temperature fluctuations during recording time were always less than 1.5°C.

### Main Laboratory Outcome Measures

**Figure 2.**
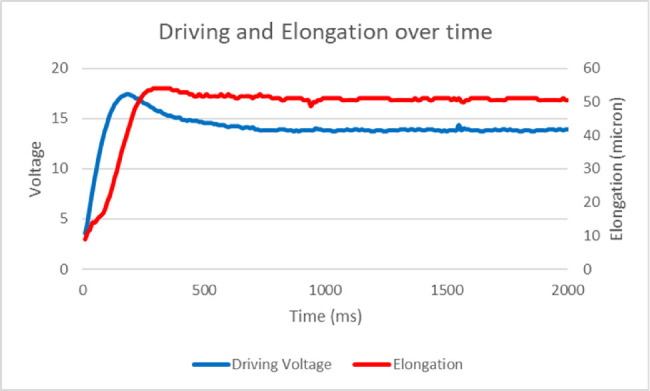
An example of DV, PV, and tip elongation as a function of time, recorded in glycerol solution (93% for this specific case). Note the accuracy of elongation (*red line*) that after a steep increase reaches the desired (50 microns) value and remains unchanged. DV (*blue line*) reaches a peak at approximately 100 milliseconds then stabilizes thanks to feedback mechanism based on PV. All different glycerol solution recordings returned similar graphs differing only for voltage intensity.

We defined DV the potential difference applied to determine phaco tip elongation and PV the potential difference generated by the direct piezoelectric effect of elongating ceramics during DV interruption and used by the feedback mechanism to match preset elongation. Right after phaco tip activation in glycerol solutions, DV showed a remarkably similar behavior regardless to preset elongation and mixture dilution: it increased initially to a peak we defined ad the maximum DV, followed by a plateau owing to feedback algorithm and constant resistance (Fig. 2). This behavior can be explained based on resistance: it takes more voltage to reach the desired elongation when the phaco tip starts moving from rest than to keep it. To calculate the resistance offered by a given glycerol dilution compared with a BSS, we defined as normalized DV (NDV) the ratio of DV in glycerol to DV in BSS. Driving tension per elongation unit was calculated as the ratio between NDV and actual phaco tip elongation expressed in microns.

### Surgical Setting and Main Surgical Setting Outcome Measures

The DV and PV data, together with all surgical settings (flow, aspiration, preset and actual tip elongation, EPT^11^) of 20 consecutive nuclear cataract cases operated on by a single experienced surgeon (TR) using the same Revo Smart phacovitrectomy machine (Optikon 2000 Inc.) were recorded as described elsewhere in this article.

Both the DV and PV were recorded continuously throughout surgery and stored in .csv files. At the beginning of surgery, the surgeon activated the primed phacoemulsificator handpiece within a test chamber filled with BSS for 2 seconds and continuous ultrasound at 25, 50, and 75 microns, respectively, to assess reference DV in BSS for proper comparison of voltage ratios referred to that specific ceramic tip setting during surgery.

Unlike laboratory tests, because of the elongation varies linearly with foot pedal depression, driving tension was calculated as the ratio of NDV to the actual elongation measured throughout the procedure rather than the preset tip elongation. In agreement with the laboratory measures described elsewhere in this article, we defined as NDV the ratio between DV per elongation unit (micron) during lens phacoemulsification and in BSS right after priming and after surgery, properly defined as:

$$\begin{array}{c} \frac{DV\left(cataract\right)}{Elongation\;Unit\;}/\frac{DV\left(BSS\right)}{Elongation\;Unit}, \end{array}$$

which equals

$$\begin{array}{c} \frac{DV_{\left(cataract\right)}}{DV_{\left(BSS\right)}}, \end{array}$$

where *DV* is the driving voltage.

The ratio of driving tensions necessary to determine a unitary elongation is a pure number, a measure of encountered resistance to phaco tip penetration into the cataractous lens, compared with that encountered by that very phaco tip during that same surgery in BSS. Nuclear cataracts opalescence and color of operated patients were graded according to LOCS III classification. The maximum and mean NDV per elongation unit correlated with the LOCS classification and EPT expressed in seconds and defined as the equivalent usage of continuous ultrasound at a 100-µm elongation.^12^

### Statistical Analyses

All continuous variables were recorded as mean ± standard deviation and analyzed by means of analysis of variance, and Pearson's two-tailed correlation coefficient was used to analyze correlation where applicable. Bivariate correlation using Pearson's correlation coefficient calculated association between variables. All tests were two-sided and the level of statistical significance was set at a *P* value of less than 0.05.

## Results

### Laboratory Setting

**Figure 3.**
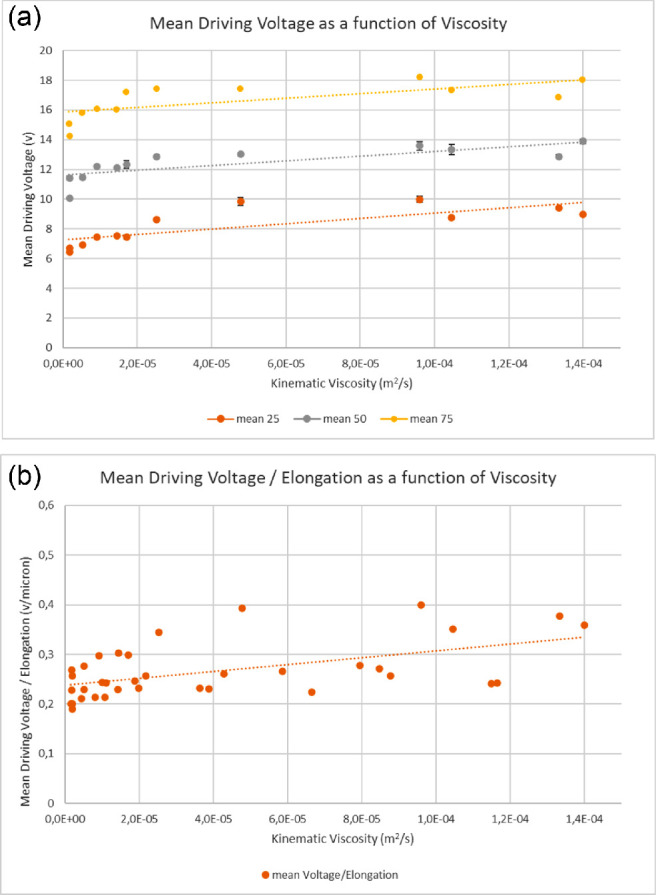
(a) Mean DV at 25, 50, and 75 microns elongation as a function of solution viscosity. Standard deviation of DV experimental points is so low that most experimental points barely show it. Note trend lines are parallel. (b) Mean DV per micron of tip elongation.

**Figure 4.**
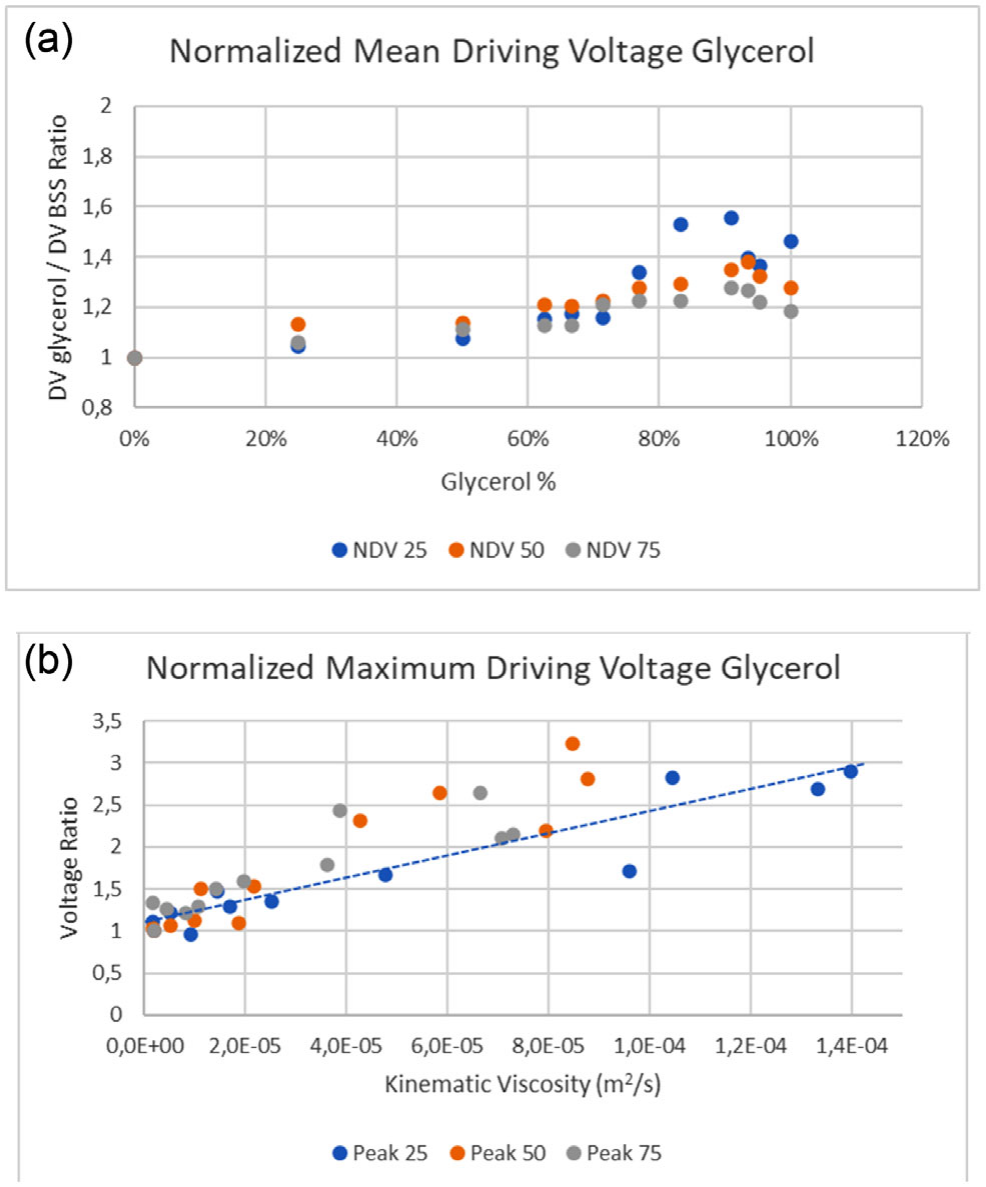
(a) Normalized mean driving tension in glycerol solution per elongation unit. *Dashed line* is the linear interpolation of data. (b) Normalized maximum driving tension in glycerol solution per elongation unit. Dashed line is the linear interpolation of data.

The mean and maximum DV significantly correlated with the glycerol solution viscosity and density at all tested elongations (*P* < 0.01 in all cases) (Table 2 and Fig. 3). The mean and maximum DV per elongation unit also linearly rose as a function of the viscosity of the mixture (*P* < 0.001) (Fig. 3b). The DV coefficient of variation, defined as (DV standard deviation)/(mean DV) of all tests in glycerol solution ranged between 0.73% and 2.78%, and the skewness factor ranged from −0.43 to 0.69, ensuring data consistency and limited dispersion with high a symmetry of tails. The n ormalized mean and maximum DV were defined as the ratio of the mean and maximum DV in glycerol solution/DV in BSS per micron of elongation (Fig. 4) also significantly correlated to solution viscosity, rising linearly at all tested elongations (*P* < 0.001).

**Table 2.** Cataract Surgery Patients Demographics, LOCS III Classification, and NDV Data

Patient	Sex	Age (y)	LOCS III Nuclear Opalesce	LOCS III Nuclear Color	EPT	Maximum	Mean	Integral	Skewness
1	F	37	2	1	2.32	3.02	1.23	1594.06	2.39
2	F	74	2	2	3.12	3.72	1.22	1736.44	3.00
3	M	75	2	3	3.20	4.08	1.30	2522.87	3.29
4	F	66	2	3	1.54	3.37	1.27	1808.66	2.11
5	F	70	3	3	1.20	3.92	1.15	1653.37	3.92
6	M	82	5	4	6.34	5.56	1.27	1870.87	4.73
7	F	88	6	5	12.00	9.32	1.27	2265.77	5.87
8	M	69	3	2	2.10	4.47	1.35	2053.60	2.40
9	M	42	1	1	1.34	2.07	1.17	80.68	0.64
10	F	91	6	5	9.22	7.58	1.35	1424.32	4.06
11	F	79	5	4	6.12	6.70	1.42	847.36	2.70
12	F	67	3	3	2.00	3.62	1.22	730.87	2.40
13	M	86	6	4	10.12	7.53	1.56	1264.02	2.73
14	F	65	3	4	2.21	5.89	1.33	646.42	2.20
15	M	75	4	4	1.54	6.74	1.29	861.21	3.86
16	F	71	3	4	1.34	5.40	1.43	981.17	2.86
17	F	76	4	3	4.54	4.29	1.22	729.76	3.01
18	M	80	4	4	7.23	5.67	1.40	1041.76	2.90
19	M	70	3	3	2.32	5.00	1.25	938.00	4.53
20	F	80	5	5	8.3	6.48	1.63	916.16	2.46

### Surgical Setting

The surgical series comprised 20 patients: 8 males and 12 females, age ranging between 37 and 91 years (mean, 72.15 ± 13.0 years), and LOCS III nuclear color and opalescence spanned the entire spectrum (1–6 and 1–5, respectively) (Table 2). The maximum NDV during surgery ranged from 2.06 to 9.32 throughout the sample and the mean NDV ranged from 1.15 to 1.62. The maximum NDV of the 20 patients sample population significantly correlated with age, EPT, LOCS III nuclear color, and opalescence (*P* < −0.001 for all cases); the mean NDV also significantly correlated to EPT, LOCS III color classification (*P* = 0.005) and opalescence (*P* = 0.018). The coefficient of variation, defined as (NDV standard deviation)/(mean NDV), ranged from 22.78% to 48.70% and the skewness between 0.30 and 5.87, indicating macroscopic data dispersion and asymmetry around the mean. NDV skewness significantly correlated with patient age (*P* = 0.004), LOCS III nuclear opalescence (*P* = 0.010), LOCS III nuclear color (*P* = 0.016), and the NDV maximum (*P* = 0.005). The integral of NDV function over time correlated significantly only to NDV data skewness (*P* = 0.026).

## Discussion

The assessment of crystalline lens hardness is a lengthy debated issue with multiple and very practical implications including the evaluation of surgical efficiency, the comparison of patients’ degree of disease, the formulation of clinical study, the validation of new operative techniques and surgical machines.^13^ The most frequently used cataract classification (LOCS III)^1^ relies on optical qualities such as nuclear color and opalescence that have been statistically associated to lens hardness in clinical studies, but remain physically unrelated to mechanical properties and indeed preclude more sophisticated analysis.

In a previous study, we demonstrated the accuracy of a patented feedback mechanism using the direct piezoelectric effect to control phaco tip elongation and vary DV^7^ according to encountered resistance. Consequently, if the DV can be precisely modulated to keep constant tip elongation against material of differing hardness, then the DV variation itself can be regarded as a measure of such material hardness.

To test this hypothesis, we started by setting up laboratory measures in glycerol dilutions offering constant, known, and increasing resistance to phaco tip oscillations, as a function of viscosity (Fig. 1) and clearly demonstrated a strict and significant correlation between DV and fluid kinematic viscosity, at all tested elongations (Figs. 3 and 4). Therefore, when a feedback mechanism returning information on tip elongation is active, DV adjustments accurately describe encountered resistance that is lens nucleus hardness variations. The existence and efficiency of such algorithm is imperative and a necessary prerequisite to establish a correlation between DV and material resistance; phacoemulsification machines not operating under such conditions cannot derive, in principle, useful information about lens hardness.

Setting a reference resistance and measure unit is the next step: different phacoemulsification handpieces equipped with innumerable tip designs, require slightly different DVs as a function of the ceramics tip inertial mass, elasticity, and resonant frequency.^14^ For this reason, we used the DV per elongation unit in BSS as the reference and the ratio of DV during phacoemulsification divided by DV in BSS during priming as a normalized pure number. As expected, this normalized measure also showed significant correlation to solution viscosity in the laboratory (Fig. 4), corroborating our hypothesis. The very limited dispersion (skewness) of NDV laboratory data witness the validity of our hypothesis given mechanic properties yield always the same NDV, which in turn increases with the resistance encountered by the tip.

**Figure 5.**
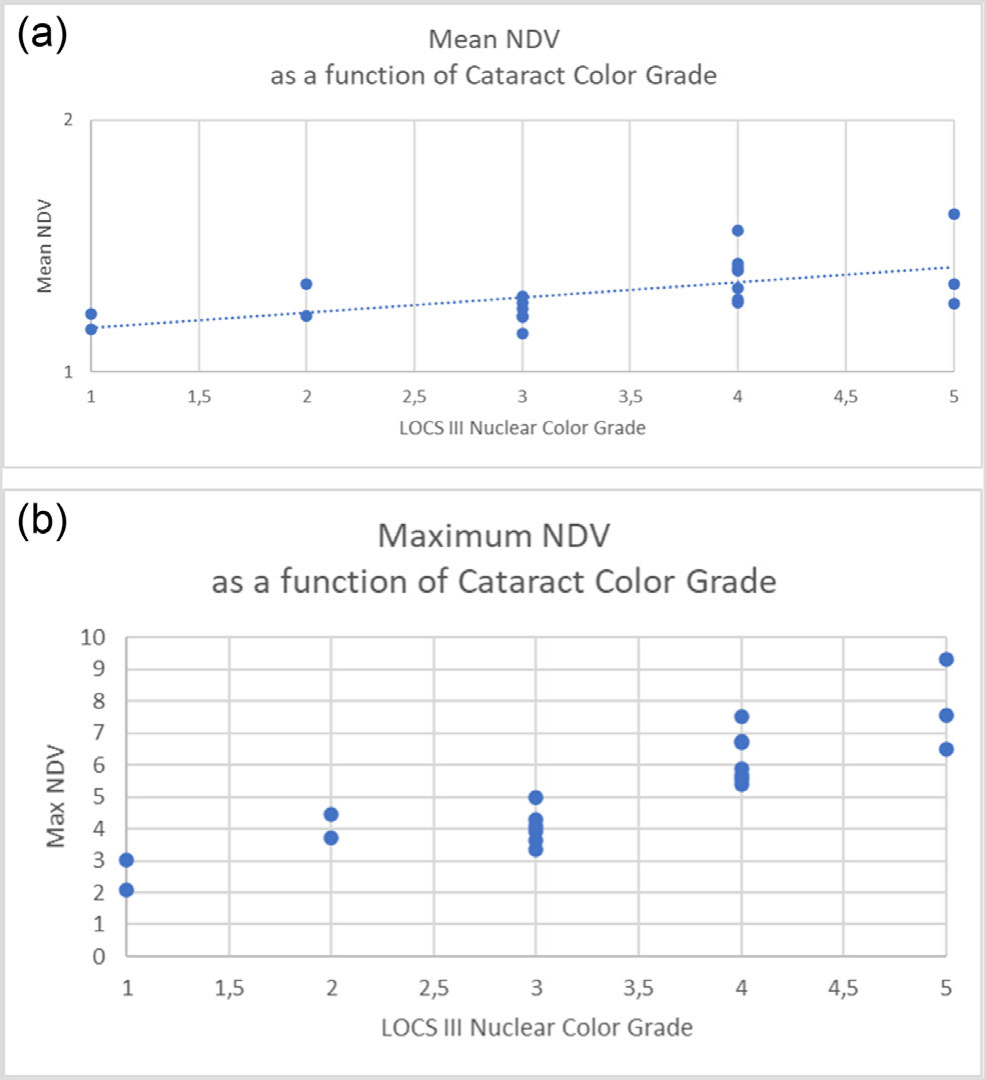
(a) Mean normalized driving tension correlation with LOCS III cataract color grade scale. The correlation is statistically significant (see text). (b) Maximum normalized driving tension correlation with LOCS III cataract color grade. The correlation is statistically significant (see text).

**Figure 6.**
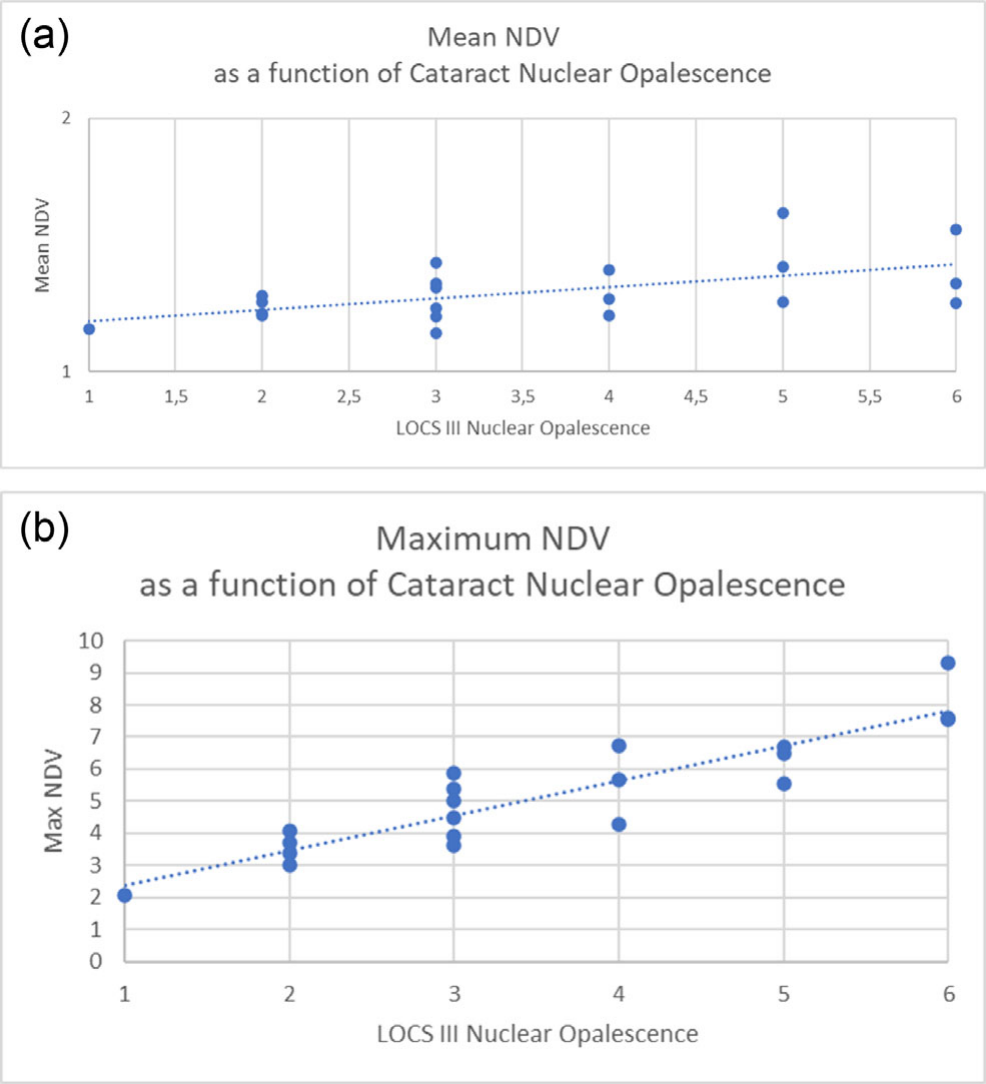
(a) Mean normalized driving tension correlation with LOCS III cataract opalescence grade scale. The correlation is statistically significant (see text). (b) Maximum normalized driving tension correlation with LOCS III cataract opalescence grade. The correlation is statistically significant (see text).

The second part of our study was aimed at assessing the reliability of NDV as a measure of the lens hardness in the operating room. Therefore, we examined the data recorded during 20 consecutive patients operated on by a single surgeon and compared NDV to LOCS III^1^ nuclear color (Fig. 5), nuclear opalescence (Fig. 6), patients’ age, NDV data skewness (Table 2), and EPT obtaining a significant correlation. This correlation provides evidence that mean and maximum NDV recorded during routine cataract surgery can discriminate lens hardness accurately and in agreement with previously validated clinical classification.

Although, from a clinical standpoint, it might be regarded as a technicality, the highly positive skewness of cataract surgery NDV data (right side of the curve) as opposed to very symmetric laboratory data, brings evidence of a double phase of materials encountered by the phaco tip during real surgery that is irrigation fluid and solid lens fragments as opposed to constant glycerol solution viscosity in the laboratory. The NDV integral function over time also proposes subtle but interesting details: it does not correlate with the LOCS III clinical color or opalescence, but only with NDV skewness, suggesting that NDV is a more accurate indicator of intraoperative lens material resistance than a clinical ophthalmoscopic assessment of transparency properties.

Although preoperative assessment of cataract hardness would undoubtedly be desirable in terms of surgical planning, the intraoperative collection of cataract resistance data may be equally important in terms of prognosis, patient staging, and surgical techniques or machine evaluation. Less obviously, but even more important, being able to assess in real time nucleus cataract hardness and its variations allows the use of the phaco tip as a probe capable of sensing cataract resistance and possibly react according to it, opening new possibilities in terms of surgical strategies and machine development.

**Figure 7.**
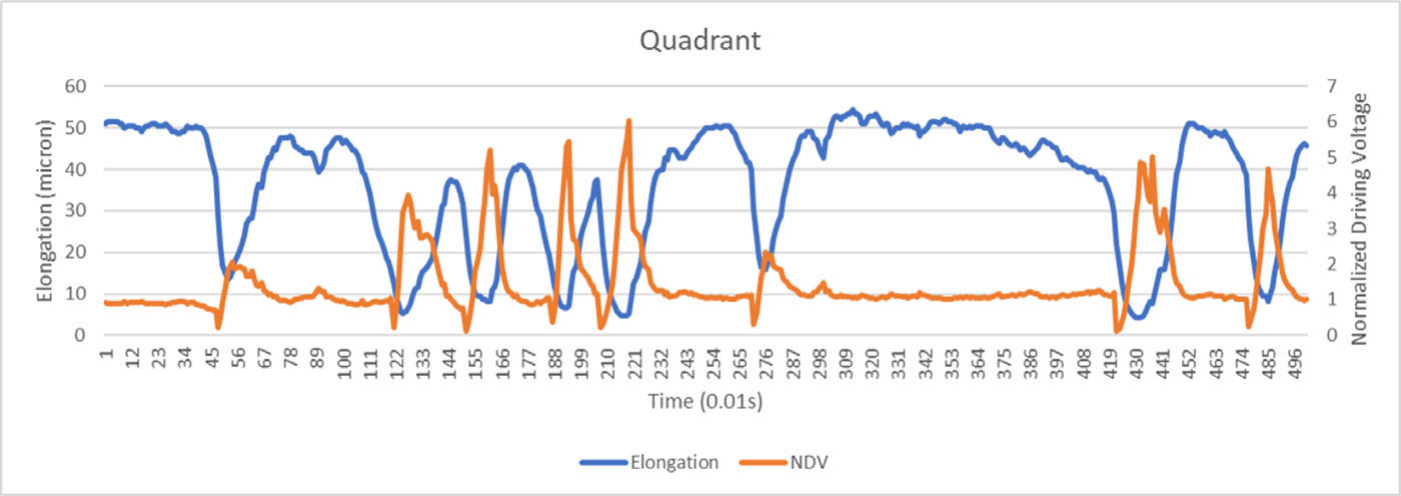
NDV and tip elongation during 5 second of phacoemulsification surgery (patient #6). Note between time 120 and 220, three consecutive NDV peaks required to reach similar elongation (around 40 microns) clearly indicate the differing resistance of encountered material necessitating a higher voltage.

As an example of intraoperative phaco tip behavior, Figure 7 shows NDV data and tip elongation recording during 5 second of phacoemulsification in quadrant removal mode with a time resolution of 0.01 seconds; note how the NDV varies continuously owing to the varying resistance of encountered lens material. This extreme resistance variability is most likely due to the layered lens anatomy and to the scalpel-like action of the tip producing debris that need to be aspirated, so that the phaco tip is alternatively jackhammering hard lens material or immersed within a slurry of fragments.

We believe our data represent proof of principle that phaco tip motion can be varied with a fuzzy logic according to real-time resistance sensing: surgical parameters, including elongation and duty cycle, could be adjusted to the purpose of minimize unnecessary ultrasound dispersion in the anterior chamber and increase efficacy. The phaco tip could conceivably idle in the sensing mode until increased resistance would signal lens material and require DV increase as needed before downgrading back to idle as soon as resistance decreases.

In summary, we took advantage of the tip elongation feedback control to infer encountered resistance and normalize DV per elongation unit with reference to BSS and successfully correlated it to the clinical cataract grading system LOCS III. The limits of the study mostly reside in the need for a feedback algorithm controlling phaco tip elongation, in the lack of which resistance variations cannot be appreciated. A larger surgical series including multiple surgeons will undoubtedly be needed.
